# Increased levels of interleukin-6 exacerbate the dystrophic phenotype in mdx mice

**DOI:** 10.1093/hmg/ddv323

**Published:** 2015-08-06

**Authors:** Laura Pelosi, Maria Grazia Berardinelli, Laura Forcina, Elisa Spelta, Emanuele Rizzuto, Carmine Nicoletti, Carlotta Camilli, Erika Testa, Angela Catizone, Fabrizio De Benedetti, Antonio Musarò

**Affiliations:** 1 Institute Pasteur Cenci-Bolognetti, DAHFMO-Unit of Histology and Medical Embryology, IIM and; 2 DAHFMO-Unit of Histology and Medical Embryology, Sapienza University of Rome, Rome 00161, Italy,; 3 Department of Mechanical and Aerospace Engineering, Sapienza University of Rome, Rome 00184, Italy,; 4 Division of Rheumatology, Bambino Gesù Children's Hospital, Rome 00100, Italy and; 5 Center for Life Nano Science@Sapienza, Istituto Italiano di Tecnologia, Rome 00161, Italy

## Abstract

Duchenne muscular dystrophy (DMD) is characterized by progressive lethal muscle degeneration and chronic inflammatory response. The mdx mouse strain has served as the animal model for human DMD. However, while DMD patients undergo extensive necrosis, the affected muscles of adult mdx mice rapidly regenerates and regains structural and functional integrity. The basis for the mild effects observed in mice compared with the lethal consequences in humans remains unknown. In this study, we provide evidence that interleukin-6 (IL-6) is causally linked to the pathogenesis of muscular dystrophy. We report that forced expression of IL-6, in the adult mdx mice, recapitulates the severe phenotypic characteristics of DMD in humans. Increased levels of IL-6 exacerbate the dystrophic muscle phenotype, sustaining inflammatory response and repeated cycles of muscle degeneration and regeneration, leading to exhaustion of satellite cells. The mdx/IL6 mouse closely approximates the human disease and more faithfully recapitulates the disease progression in humans. This study promises to significantly advance our understanding of the pathogenic mechanisms that lead to DMD.

## Introduction

Duchenne muscular dystrophy (DMD) is an X-linked genetic disease caused by mutations in the dystrophin gene (1). The mdx mouse strain, lacking a functional dystrophin gene, has served as the animal model for human DMD (2). However, while the skeletal muscles of mdx mice undergo extensive necrosis early in neonatal life, the affected muscle rapidly regenerates and regains structural and functional integrity (3–7). The basis for the mild effects of the absence of dystrophin observed in mice compared with the lethal consequences in humans remains unknown. Among factors possibly explaining the difference between humans and mdx mice, the extent of chronic inflammatory response has been suggested to be linked to the severity of dystropathology (8,9).

In the present study we have focused our attention on the inflammatory cytokine interleukin-6 (IL-6), based on the evidences that (i) IL-6 increases in DMD patients compared with healthy subjects (10–12), (ii) high levels of IL-6 promote muscle proteolysis (13–15), (iii) selective modulation of inflammatory response ameliorates muscle pathology in mdx mice, reducing IL-6 (16) and (iv) inhibition of the IL-6 activity (17) and of the intracellular mediator, namely the JAK/STAT pathway, stimulates muscle regeneration in both aged and dystrophic mice (18,19). IL-6 is a pleiotropic cytokine that is produced by different cell types and has the capacity to induce several different intracellular signaling pathways (20). IL-6 exerts its biological activities through two receptors: the membrane-bound and the soluble IL-6 receptors. The membrane-associated IL-6 receptor on target cells forms a heterodimer with the cell surface gp130 receptor, and this complex activates intracellular signaling pathways known as classic-signaling (21). IL-6 trans-signaling requires soluble IL-6R (sIL-6R) and is possible on all cells of the body since all cells express the gp130 protein (22).

Based on the activation of either classic or trans-signaling, IL-6 can promote markedly different cellular responses. Of note, IL-6 trans-signaling is pro-inflammatory, whereas classic IL-6 signaling promotes regenerative or anti-inflammatory activities of the cytokine (21).

Circulating IL-6 levels are normally very low or undetectable and are markedly increased in several diseases associated with inflammation, inducing the transition from an acute to a chronic inflammatory response (23). Circulating IL-6 is also well described as predictor of weight loss in human cancer cachexia (20,24,25) and it contributes to a pathologic state associated with aging called inflamm-aging (26). IL-6 is also locally and transiently produced in response to exercise and following injury, coinciding with the active period of muscle regeneration, and it plays an important role in satellite cell proliferation, muscle growth, glucose uptake and fat oxidation (27–29). In contrast, increased muscle proteolysis was found after administration of high doses or long-term exposure to IL-6 in rodents (13–15). We demonstrated that treatment of C2C12 myogenic cells with recombinant IL-6 resulted in a dramatic inhibition of myoblasts differentiation (30). Moreover, we recently described the success of pharmacological inhibition of IL-6 activity, by use of a neutralizing antibody against the IL-6 receptor, in mdx dystrophic mouse model at the age in which skeletal muscle is severely compromised and at the stage when the manifestations of the disease in the mdx mouse model are clearly evident (17). It has been also reported that treatment of mdx with either Compound A, a non-steroidal selective glucocorticoid receptor modulator or with amitriptyline, a tricyclic antidepressant drug, improved muscle function and decreased serum creatine kinase (CK) levels and reduced pro-inflammatory cytokines expression, including IL-6 (16,31).

The conflicting activity of IL-6 and the apparent discrepancy among different studies can be justified considering that the positive effects of IL-6 are normally associated with its transient production and short-term action (20,32,33) and that IL-6 might exert opposite roles in different cell types. In addition, a chronic elevation of circulating IL-6 is often associated with an underlying disease, and specific aspects of different disease states could serve to alter the systemic and tissue level response to elevated IL-6.

To add new insights into the pathogenesis of muscular dystrophy and to determine the basis for the milder phenotype observed in adult mdx mice, compared with young mice and to human patients, we crossed the mdx mice with IL-6 overexpressing mice, which accumulate high levels of circulating IL-6 since early phases of life (34). In the present study, we found that IL-6 was indeed expressed at higher levels in the diaphragm of 4-week-old mdx mice compared with 24-week-old mdx animals, possibly suggesting that a down-regulation of this cytokine might contribute to an amelioration of dystrophic phenotype observed in the adult (3). Increased levels of IL-6 induced, in mdx/IL6 mice, the severe features and progressive nature of human DMD, in a stage normally spared by the absence of dystrophin, suggesting that IL-6 is causally linked to the pathogenesis of muscular dystrophy.

## Results

### Down-regulation of inflammatory cytokines correlates with amelioration of the dystrophic phenotype in mdx mice

**Figure 1.**
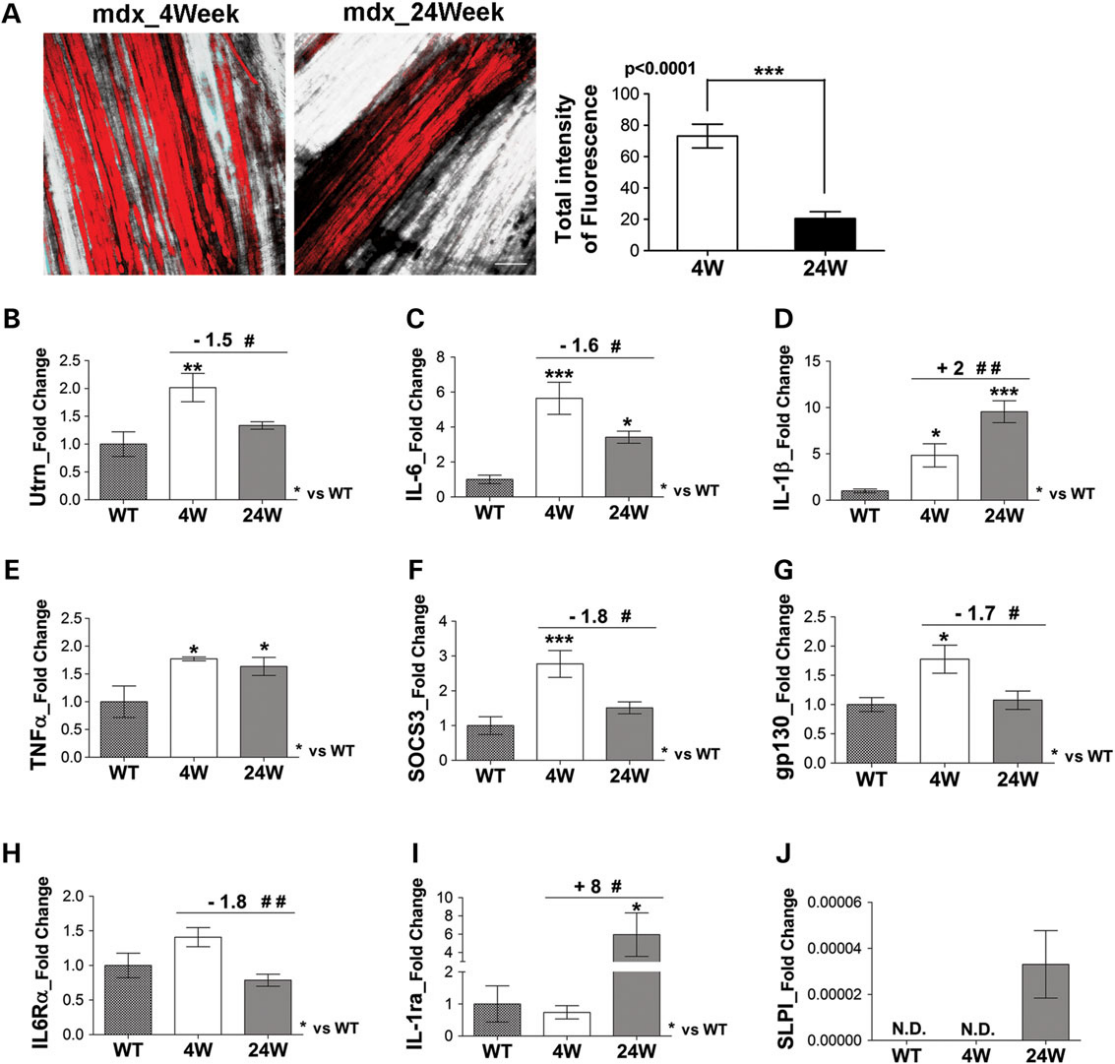
The down-modulation of IL-6 correlates with the stabilization of dystrophic phenotype. (**A**) Confocal microscopy (left panels): representative images of Evans Blue Dye (EBD) staining of whole diaphragm muscles from mdx mice at 4 weeks (4W, *n* = 5) and 24 weeks (24W, *n* = 4) of age. The images show necrotic fibers in red and optical phase in white. Scale bar, 200 μm. Graph showing quantification of EBD uptake (right panel) performed on 160–210 optical sections with a step size of 10 μm, from whole diaphragms of indicated genotypes. EBD is significantly more diffuse (72%) in the diaphragm of 4-week-old mdx than in the diaphragm of 24-week-old mdx mice. Values represent mean ± SEM. *P* value by Mann–Whitney test. (**B**–**J**) Real time PCR analysis performed on diaphragm muscles from wild-type (WT) and mdx mice at 4 and 24 weeks of age, for the expression of Utrn (B), IL-6 (C), IL-1β (D), TNFα (E), SOCS3 (F), gp130 (G), IL6Rα (H), IL-1ra (I) and SLPI (J). Values represent mean ± SEM; *n* = 4–9 mice per group. ***P < 0.0005, **P < 0.005, *P < 0.05, compared with WT mice; ^##^*P* < 0.005 ^#^*P* < 0.05 between 4-week-old and 24-week-old mdx mice (by ANOVA).

A pathogenic feature associated with the severity of dystropathology is the necrosis of muscle fibers, accompanied by inflammatory response (35,36). Necrosis was significantly reduced in the diaphragm muscle of 24-week-old mdx mice compared with 4-week-old mdx mice (Fig. 1A). Of note, we also observed a significant reduction in utrophin (Utrn) expression in the diaphragm of 24-week-old mdx mice compared with 4-week-old mdx mice (Fig. 1B), further supporting the hypothesis of activation of compensatory mechanism in adult dystrophic muscle (37,38), leading to sarcolemma integrity. The reduced damage in the diaphragm of adult mdx mice would also suggest a modulation of the inflammatory response, which is a prominent feature of the pathogenesis of muscular dystrophy (9,39). We analysed the expression levels of IL-6, TNFα and IL-1β, important mediators of the inflammatory response (40,41), in the diaphragm of mdx mice at 4 and 24 weeks of age. Real time PCR analysis revealed that the expression of IL-6 (Fig. 1C), IL-1β (Fig. 1D) and TNFα (Fig. 1E) was significantly up-regulated in diaphragm of 4-week-old mdx mice, compared with wild-type littermates. Among these cytokines, IL-6 was the only one significantly down-regulated in the diaphragm muscle of 24-week-old mdx mice, compared with 4-week-old mdx mice (Fig. 1C). The down-regulation of IL-6 in the diaphragm of 24-week-old mdx mice was also associated with the reduced expression of SOCS3 (Fig. 1F), a negative regulator of IL-6 signaling (42,43), and of gp130 (Fig. 1G) and IL6Rα (Fig. 1H), the two IL-6 receptors that mediate the IL-6 signaling (22).

To further support the evidence of reduced inflammatory response in the diaphragm muscle of 24-week-old mdx mice, compared with 4-week-old mice, we evaluated relevant markers of the anti-inflammatory response, such as IL-1ra and SLPI. Real time PCR analysis revealed a significant up-regulation of IL-1ra (Fig. 1I) and SLPI (Fig. 1J) expression in the diaphragm of adult mdx mice.

These data are consistent with the hypothesis that the down-modulation of IL-6 signaling might be sufficient to mitigate the inflammatory response in dystrophic muscles and, possibly, to attenuate the muscle phenotype observed in adult mdx mice.

### IL-6 overexpression increases the number of regenerating centro-nucleated fibers in mdx mice

In order to evaluate the potential role of IL-6 overexpression on adult dystrophic phenotype, we generated the mdx/IL6 mouse model, by crossing mdx mice with IL-6 transgenic mice that express high levels of circulating IL-6 since early after birth (34).

**Figure 2.**
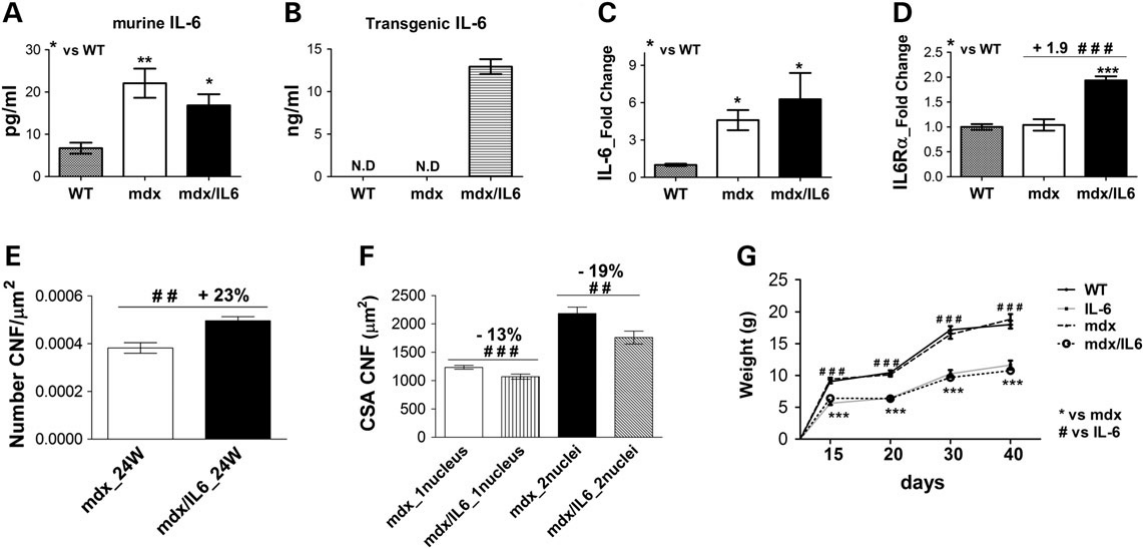
Increased levels of IL-6 enhance the number of regenerating centro-nucleated fibers in mdx mice. (**A** and **B**) Circulating levels of endogenous IL-6 (A) and of transgenic IL-6 (B) was evaluated by ELISA on serum from 24-week-old wild-type (WT), mdx and mdx/IL6 mice. Values represent mean ± SEM; *n* = 5–10 mice per group. **P < 0.005 between 24-week-old mdx mice and WT littermates; *P < 0.05 between 24-week-old mdx/IL6 and WT mice (by *t*-test). *ND*, no detected. (**C** and **D**) Real time PCR analysis for the expression of IL-6 (C) and IL6Rα (D) in diaphragm of 24-week-old WT, mdx and mdx/IL6 mice. Values represent mean ± SEM; *n* = 4–8 mice per group. *P < 0.05 compared with WT littermates (by *t*-test for panel C); ***P < 0.0005 compared with WT mice, ^###^*P* < 0.0005 between mdx/IL6 and mdx mice (by ANOVA for panel D). (**E** and **F**). Quantification of total number of CNFs per μm^2^ (E) and of the average cross sectional area (CSA) of CNFs with 1 and 2 nuclei (F) in muscles of 24-week-old mdx and mdx/IL6 mice. Values represent mean ± SEM; *n* = 5 mice per group. ^###^*P* < 0.0005, ^##^*P* < 0.01 using Mann–Whitney test. (**G**) Growth curves of wild-type (WT, *n* = 17), IL-6 transgenic (IL-6, *n* = 5), mdx (*n* = 8) and mdx/IL6 (*n* = 16) mice. Values represent mean ± SEM. ^###^*P* < 0.001 between IL-6 transgenic and WT mice; ***P < 0.001 between mdx/IL6 and mdx mice using two-way ANOVA.

We evaluated, by ELISA, both endogenous and transgenic plasma circulating levels of IL-6 in wild-type, mdx and mdx/IL6 mice of 24 weeks of age (Fig. 2A and B). As expected, the endogenous levels of circulating IL-6 were very low in wild-type mice and increased in both mdx and mdx/IL6 mice (Fig. 2A). Nevertheless, we did not observed any significant change in endogenous circulating levels of IL-6 between mdx and mdx/IL6 mice (Fig. 2A). In contrast, ELISA revealed a dramatic increase of circulating levels of transgenic IL-6 in mdx/IL6 mice, compared with both wild-type and mdx mice (Fig. 2B). We also evaluated the expression levels of IL-6 in the diaphragm of wild-type, mdx and mdx/IL6 mice. The levels of muscle expression of IL-6 were increased in both mdx and mdx/IL6 mice of 24 weeks of age, compared with wild-type littermates (Fig. 2C); however there was no statistical difference in the expression levels of muscle IL-6 between mdx and mdx/IL6 mice (Fig. 2C). Interestingly, IL-6 receptor alpha, which mediates the IL-6 trans-signaling, was significantly up-regulated in the muscle of 24-week-old mdx/IL6 mice, compared with both wild-type and mdx littermates (Fig. 2D).

To determine whether increased levels and activity of IL-6 exacerbate the dystrophic muscle phenotype, a direct comparison of morphological parameters was performed for diaphragm muscle of mdx and mdx/IL6 mice at 24 weeks of age. The analysis revealed a significant increase in the number of small centrally nucleated fibers (CNFs) (Fig. 2E), which represents a morphological feature of newly regenerating fibers and an indication of ongoing regeneration (39), in the muscle of 24-week-old mdx/IL6 mice. Notably, the average cross-sectional area (CSA) of centro-nucleated fibers with 1 and 2 nuclei were also significantly smaller in the muscle of 24-week-old mdx/IL6 mice, compared with mdx littermates (Fig. 2F). Interestingly, CSA of both regenerating and not-regenerating myofibers was smaller in diaphragm muscle of the 24-week-old mdx/IL6 mice compared with mdx littermates (data not shown), suggesting that IL-6 negatively influences the myofiber size of dystrophic muscle. Since the reduced myofiber CSA in mdx/IL6 diaphragm could depend on the IL-6 smaller mouse (34) and in order to verify whether IL-6 overexpression affects the growth rate of mdx mice, we analysed and compared the growth curves of wild-type, IL-6 transgenic, mdx and mdx/IL6 mice (Fig. 2G). We observed that elevated levels of circulating IL-6 induced a similar growth defect in both IL-6 and mdx/IL6 mice (Fig. 2G), compared with wild-type and mdx mice. This indicates that muscular dystrophy does not further increase the effects of IL-6 on growth defect. To confirm these data we calculated the muscle weight/body weight ratio in wild-type, IL-6 transgenic, mdx and mdx/IL6 mice at 24 weeks of age (Table 1). As shown in Table 1, the significant reduction of diaphragm mass in mdx/IL6 mice was related to the decrease of the body weight, leading to a similar muscle weight /body weight ratio between mdx/IL6 and mdx mice.

**Table 1.** Body weight and diaphragm mass of wild-type, IL-6 transgenic, mdx and mdx/IL6 mice at 24 weeks of age

	Wild type	*P* Value	IL-6	*P* Value	mdx	*P* Value	mdx/IL6	*P* Value
		versus IL-6		versus mdx/IL6		versus WT		versus mdx
Body weight (g)	35.81 ± 7.71	0.0005	23.22 ± 4.02	ns	37.93 ± 6.44	ns	28.59 ± 3.70	0.005
Diaphragm weight (g)	0.09 ± 0.02	0.005	0.07 ± 0.01	0.0005	0.16 ± 0.04	0.0005	0.13 ± 0.03	0.05
Muscle weight/Body weight ratio	0.0026 ± 0.0005	ns	0.0028 ± 0.0006	0.0005	0.0041 ± 0.0007	0.0005	0.0044 ± 0.0008	ns

*Note:* Data represent the means ± SD of at least nine animals per group. IL-6 = IL-6 transgenic mouse. *P* values by one-way ANOVA. ns = not significant.

These data reveal that: (i) increased circulating levels of IL-6 cause growth defect in IL-6 transgenic and mdx/IL6 dystrophic mice, (ii) IL-6 sustains a continuous cycle of muscle regeneration when overexpressed in the context of muscular dystrophy.

### Increased levels of IL-6 enhance the susceptibility of dystrophic muscle to damage

We then performed experiments to substantiate the hypothesis that increased levels of IL-6 exacerbate the dystrophic phenotype and negatively influence the lifespan of mdx mice.

**Figure 3.**
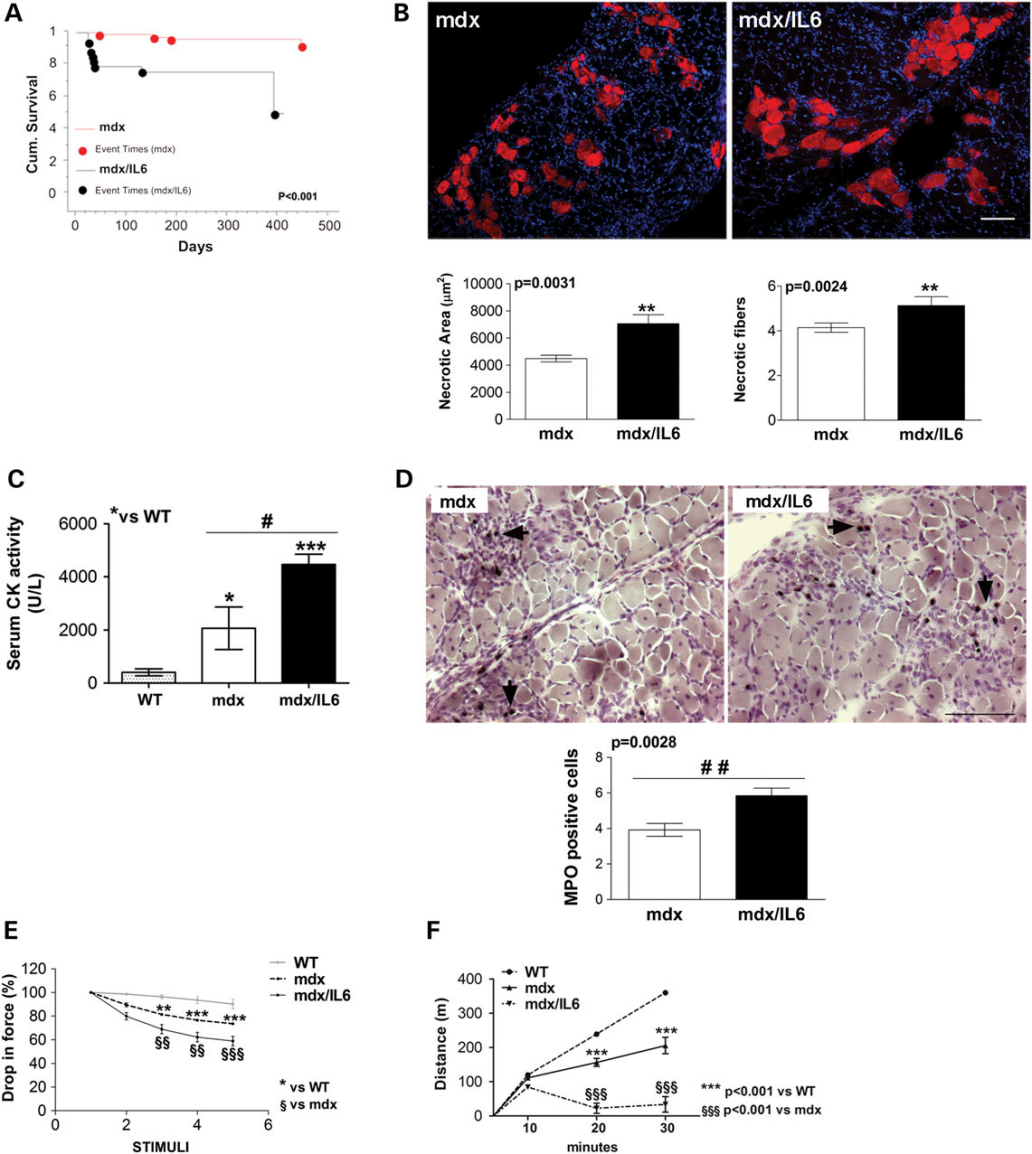
IL-6 overexpression exacerbates the dystrophic phenotype. (**A**) Kaplan–Meier survival curves of indicated genotypes. *n* = 39–98 mice per group. *P* value compared with mdx mice using long-rank test. (**B**) Top panels: Representative images of Evans blue dye (EBD) staining of transverse section of diaphragm from 24-week-old mdx and mdx/IL6 mice. Bottom panels: quantification of necrotic areas and of Evans-blue-dye positive cells within necrotic area (necrotic fiber) in diaphragm of 24-week-old mdx and mdx/IL6 mice. Scale bar, 100 μm. Values represent mean ± SEM; *n* = 5 mice per group. *P* values, compared with mdx mice using Mann–Whitney test. (**C**) Serum CK levels in 24-week-old mice of indicated genotypes. Values represent mean ± SEM; *n* = 6–7 mice per group. ***P < 0.0005, *P < 0.05 compared with WT littermates; ^#^*P* < 0.05 between mdx/IL6 and mdx mice using one-way ANOVA. (**D**) Representative image of MPO staining (top panels) of transverse section of diaphragm from 24-week-old mdx and mdx/IL6 mice. Bottom panel shows the evaluation of the number of phagocytic cells (MPO-positive-cells) in diaphragm of 24-week-old mdx and mdx/IL6 mice. Scale bar, 100 μm. MPO^+^ cells (black arrows) are much more in number in the diaphragm of mdx/IL6 than in the diaphragm of mdx mice. Values represent mean ± SEM; *n* = 5 per group. *P* value compared with mdx mice using Mann–Whitney test. (**E**) The graph shows the progressive drop in force during five subsequently electrical stimulations (stimuli) in EDL muscle from WT, mdx and mdx/IL6 mice of 24 weeks of age. Values represent mean ± SEM; *n* = 5–6 mice per group. **P < 0.01, ***P < 0.001 compared with WT littermates; ^§§^*P* < 0.01, ^§§§^*P* < 0.001 compared with mdx mice using two-way ANOVA. (**F**) Quantification of treadmill test on wild-type (WT), mdx and mdx/IL6 mice of 24 weeks of age. The graph shows the covered distance, by indicated genotypes, in a given time at 10, 20 and 30 min of the running test. Values represent mean ± SEM; *n* = 3 mice per group. *P* value using two-way ANOVA.

Of note and conversely to the DMD patients, the lifespan of mdx mice is comparable to healthy wild-type mice (44). We observed that high levels of circulating IL-6, in the context of the dystrophic genotype, significantly reduced the survival of mdx/IL6 mice by 100 days to a maximal life span of 400 days (*P* < 0.001), compared with mdx mice (Fig. 3A).

One of the pathogenic events associated with DMD is the continuous cycle of degeneration and regeneration (4). The presence of small regenerating fibers in the muscle of 24-week-old mdx/IL6 mice would reflect an active myofibre necrosis. We thus evaluated, by Evan's blue dye staining (EBD), the degree of muscle degeneration (45) in the diaphragm muscle of 24-week-old mdx and mdx/IL6 mice (Fig. 3B). The areas and the number of necrotic fibers within the injured area (Fig. 3B bottom panel) were significantly larger in the diaphragm of mdx/IL6 mice than in the mdx littermates. Another feature of muscle degeneration is the release of muscle enzymes, generally assayed as CK, into the circulation (46). We found a significant increase of serum CK levels in 24-week-old mdx/IL6 mice compared with mdx littermates (Fig. 3C).

Muscle damage leading to regeneration, is normally associated to activation of the inflammatory response (39). Histological and histochemical analysis revealed an accumulation of infiltrating mononuclear cells (Fig. 3D top panels) and an increased concentration of myeloperoxidase (MPO)-positive cells (Fig. 3D bottom panels) within the muscle of 24-week-old mdx/IL6 mice, compared with mdx muscle. The damage of muscle fibers might compromise muscle force-producing capacity. We evaluated the EDL muscle function, namely the capacity to produce force, of 24-week-old wild-type, mdx and mdx/IL6 mice. The mdx/IL6 muscle showed significantly greater drop in force between the first and fifth contractions compared with mdx and wild-type littermates (Fig. 3E). To define, in a more physiopathological context, the alteration in the functional performance of mdx/IL6 dystrophic muscle, we forced mice to run on a treadmill, which is also used to accelerate muscle weakness to more closely mirror the muscle pathology seen in patients with DMD (47–51).

We applied a protocol of 30 min running on a horizontal motorized treadmill, equipped with a grid, at a speed of 12 m/min, twice a week for 5 weeks (49), starting at 24 weeks of age. We revealed a significant reduction in the functional performance of mdx/IL6 mice, compared with mdx and wild-type littermates (Fig. 3F). In particular, the mdx/IL6 mice covered a minor distance compared with mdx mice littermates (Fig. 3F). We noted that the mdx/IL6 mice received more stimuli to run (either electric foot shock or tongue depressor stimulation) and remained on the grid for an extended period of time compared with mdx littermates. The mdx/IL6 mice were exhausted after the exercise session and remained prostrated in the cages for an extended period of time compared with mdx littermates.

### Molecular mediators of IL-6 pathologic activity in dystrophic muscle

**Figure 4.**
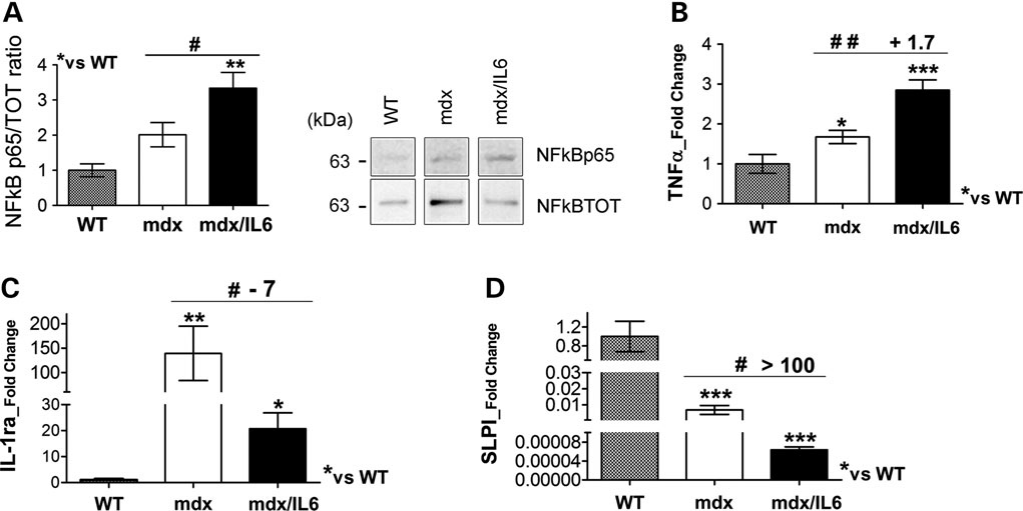
Molecular mediators of dystrophic muscle by IL-6 overexpression. (**A**) Western blot (right panel shows a representative image) analysis for NFkB active (NFkB p65) and total (NFkB TOT) proteins, performed on diaphragm of 24-week-old WT, mdx and mdx/IL6 mice. Values represent mean ± SEM; *n* = 5–10 per group. **P < 0.005 compared with WT mice; ^#^*P* < 0.05 between mdx/IL6 and mdx littermates (by one-way ANOVA). (**B**–**D**) Real time PCR analysis for the expression of TNFα (B), IL-1ra (C) and SLPI (D) on diaphragm of indicated genotypes at 24 weeks of age. Values represent mean ± SEM; *n* = 4–7 per group. ***P < 0.0005; **P < 0.005; *P < 0.05 compared with WT mice; ^##^*P* < 0.005; ^#^*P* < 0.05 between mdx/IL6 and mdx littermates (by one-way ANOVA). In A the lanes were run on the same gel but were non contiguous.

One of the central players implicated in inflammation and muscle wasting and a pathogenic factor in DMD is NFkB (52–55). Western blot analysis revealed a significant increase of NFkB phosphorylated active form (Fig. 4A) in the diaphragm from 24-week-old mdx/IL6 mice compared with mdx littermates. Notably, the expression levels of NFkB were not significantly different in the muscles of 24-week-old wild-type and mdx mice (Fig. 4A). TNFα expression (Fig. 4B), a potent NFkB inducer and a cytokine that mediates skeletal muscle protein degradation (56,57) was up-regulated in the muscle of both 24-week-old mdx and mdx/IL6 mice compared with wild-type littermates. Of note, TNFα expression resulted significantly up-regulated in the diaphragm of mdx/IL-6 compared with mdx littermates (Fig. 4B). These data further support the evidence that the mild muscle phenotype of adult mdx mice is due to the reduction in the inflammatory response, whereas the inflammatory infiltrate that characterize the muscle of mdx/IL6 mice might play a major role in promoting the pathology of dystrophin-deficient muscle. Indeed, the enhanced inflammatory response and the exacerbated dystrophic phenotype of mdx/IL6 mice were also associated with a significant down-regulation of the anti-inflammatory modulators IL-1 receptor antagonist (IL-1ra) (Fig. 4C) and of SLPI (Fig. 4D) (58,59).

### Increased levels of IL-6 contribute to the exhaustion of muscle stem cells (MuSCs)

**Figure 5.**
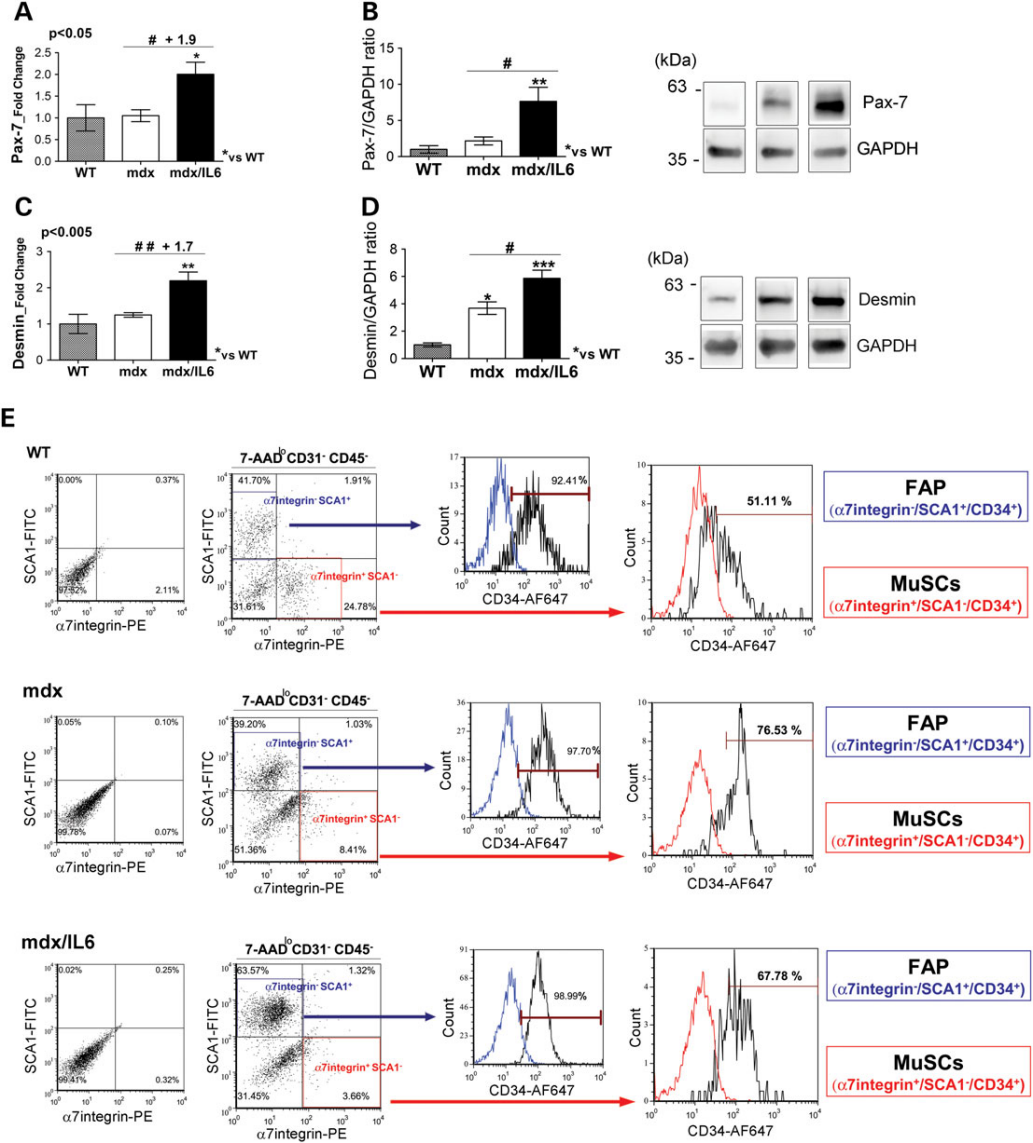
Increased levels of IL-6 induce exhaustion of MuSCs and accumulation of FAPs. (**A** and **C**) Real time PCR analysis for the expression of Pax-7 (A) and Desmin (C) on diaphragm of 24-week-old wild-type (WT), mdx and mdx/IL6 mice. Values represent mean ± SEM; *n* = 5–7 per group. *P* values using one-way ANOVA. (**B** and **D**) Densitometric analysis (right panels) and representative images (left panels) of western blot analysis for the expression of Pax-7 (B) and Desmin (D) on diaphragm of 24-week-old wild-type (WT), mdx and mdx/IL6 mice. Values represent mean ± SEM; *n* = 5–9 per group. ***P < 0.0005; **P < 0.005; *P < 0.05 compared with WT mice; ^#^*P* < 0.05 between mdx/IL6 and mdx littermates (by ANOVA). (**E**) Cytofluorimetric analysis of freshly isolated cells from muscles of 36-week-old wild-type (WT), mdx and mdx/IL6 mice. Living cells were gated for 7-AAD low. Cells negative for CD45 and CD31, markers of macrophages, hematopoietic and endothelial cells, were gated and analysed for the expression of Sca1 and α7-integrin. Within α7-integrin^+^/Sca1^−^ population (red gate) the muscle satellite cells (MuSCs) were identified as lin^−^/α7-integrin^+^/Sca1^−^/CD34^+^ cells. α7-integrin^−^/Sca-1+ cells (blue gate) identified fibro/adipogenic progenitors (FAPs) and over 92% of these cells expressed CD34. *n* = 4 independent experiments. *P* < 0.05 compared with mdx littermates using student's two-tailed *t*-test*.* In B, D the lanes were run on the same gel but were non contiguous.

One of the pathological features of the severe dystrophic phenotype is a continuous cycle of degeneration and regeneration that is exacerbated in mdx mice by increased levels of IL-6. To support morphological analysis (Fig. 2E and F; Fig. 3) and verify the behavior of satellite cells, we evaluated the expression of Pax-7, a marker of quiescent and activated satellite cells (60,61) and Desmin, a specific marker of satellite cells activation and proliferation (62,63). The transcripts (Fig. 5A and C) and proteins (Fig. 5B and D) of both Pax-7 and Desmin resulted significantly up-regulated in the muscle of 24-week-old mdx/IL6 mice compared with mdx littermates. These data suggest that increased levels of circulating IL-6 interfere with muscle homeostasis and stimulate a continuous satellite cells proliferation (64), eventually leading to exhaustion of the pool of MuSCs.

To validate this hypothesis, we analysed, in both 36-week-old mdx and mdx/IL6 mice, the percentage of MuSCs. Accordingly to previous work, the percentage of MuSCs were determined by flow cytometry as CD34^pos^/α7-integrin^pos^/Sca1^neg^/CD45^neg^/CD31^neg^ cells (65). CD34^pos^/α7-integrin^pos^ cell population was significantly reduced in the muscle of mdx/IL6 mice compared with mdx littermate (Fig. 5E). Because the combination of CD34 and α7-integrin expression is limited to quiescent self-renewal MuSCs with myogenic activity (65,66), the reduction in this population suggests that accumulation of circulating IL-6 levels causes an exhaustion of MuSCs. Of note, cytofluorimetric profile revealed also an increase in the percentage of lin^neg^/Sca1^pos^/CD34^pos^/α7-integrin^neg^ cells. These cells have been recently described as fibro/adipogenic progenitors (FAPs), which modulate muscle regeneration in healthy muscle and contribute to fibrosis under pathologic conditions (66–71). Our data suggest that increased levels of IL-6 create an hostile microenvironment that affects MuSCs maintenance and exacerbates the dystrophic phenotype.

## Discussion

In this study, we contributed to define the pathogenic role of IL-6 in the exacerbation of the dystrophic phenotype in mdx dystrophic mouse model.

A longstanding open question in the field of muscular dystrophy has been why DMD patients display a severe muscle wasting, whereas the mdx mouse strain, with the same mutation, has a nearly normal lifespan and does not develop severe phenotype (3). The basis for the mild effects observed in mice compared with the lethal consequences in humans remains unknown. In our study, we addressed whether a critical component of the immune system, namely the pro-inflammatory cytokines IL-6, plays a pivotal role in the pathogenesis of DMD.

The rationale to study the role of IL-6 on the pathogenesis of muscular dystrophy was based on the evidence that: (i) the inflammatory cytokine IL-6 concentrations are significantly higher in the serum of DMD patients compared with healthy age-matched (12,17); (ii) IL-6 concentrations follow the disease time-course in human DMD (12); (iii) among pro-inflammatory cytokines, IL-6 was the only one significantly down-regulated in the muscle of 24-week-old mdx mice (Fig. 1), compared with younger mice, which represents a stage normally spared from the absence of dystrophin (7,72,73).

We postulated that the mild muscle wasting phenotype observed in adult (24-week-old) mdx mice, compared with young (4-week-old) mdx mice is due to the reduction in IL-6 expression. To substantiate this hypothesis, mdx mice and IL-6 transgenic mice (34), which accumulate IL-6 in the blood since birth, were used to generate the mdx/IL6 transgenic animals. The mdx/IL6 mice displayed a dramatic and selective increase of circulating levels of transgenic IL-6, compared with both wild-type and mdx mice.

Our data in mdx/IL6 mice, show that increased circulating levels of IL-6 cause reduction in survival of dystrophic mouse model, increase muscle necrosis and inflammation, sustain repeated cycles of muscle degeneration and regeneration and contribute to exhaustion of satellite cells, leading to exacerbation of the dystrophic muscle phenotype. Therefore, we provided evidences demonstrating that sustained increase in the levels of IL-6 alone is sufficient to exacerbate the dystrophic phenotype at a stage, 24 weeks of age, when only a mild muscle phenotype is apparent in mdx mice (3). This indeed complements the observation of a selective down-regulation of IL-6 in the diaphragm of 24-week-old mdx mice compared with 4-week-old mdx mice.

We then characterized the potential molecular pathways that mediate the catabolic effects of increased levels of IL-6. IL-6 exhibits pro-inflammatory activity through activation of the transcription factor nuclear factor *κ*B (NFkB), which represents also a key mediator of muscle wasting in different pathologic conditions, including DMD (74,75). We observed a significant up-regulation of NFkB and TNFα expression, potent inducer of NFkB and a cytokine that mediates skeletal muscle protein degradation (56,57), in the diaphragm of 24-week-old mdx/IL6 mice.

Another pathogenic event associated with DMD is the exhaustion of satellite cells that replace damaged fibers, due to a continuous process of degeneration and regeneration. The persistent activation of satellite cells observed in mdx/IL6 muscles supports the evidence that IL-6 is an important mediator of satellite cell proliferation (64). We reasoned that the repeated cycles of degeneration/regeneration, induced by IL-6 overexpression, might contribute to the exhaustion of MuSCs) and, therefore, to the exacerbation of the dystrophic phenotype. Indeed, cytofluorimetric profile revealed a significant reduction of MuSCs in mdx/IL6 muscles compared with mdx one, associated with an increase in Sca1^pos^ fibro/adipocytic infiltration. This suggests that increased levels of IL-6 generate an unfavorable microenvironment, which stimulates fibroadipogenic progenitors (FAPs) proliferation/activity. In the context of healthy muscle, FAPs provide an environment that favors myogenic differentiation (66). However, in chronic injury, such as muscular dystrophy, FAPs contribute to disease progression by influencing the activity of satellite cells (66–68). Interestingly, FAPs represent an inducible source of IL-6 (66). Although we specifically analysed the effect of increased levels of IL-6 on FAPs population, we cannot exclude an alteration in the activity of other precursor cells, which present a similar molecular signature of FAPs, namely mesoangioblasts, PW1+ interstitial cells (PICs) and Side population (SP) cells (76–78), whose alteration can in turn hinder FAPs function and muscle homeostasis. Of note, our work also supports recent studies, demonstrating that elevated Stat3, a down-stream effector of the pro-inflammatory cytokine IL-6, inhibits satellite cell function and impair regeneration during aging and disease progression (18,19).

One open question is whether IL-6 directly targets skeletal muscle or alternatively works through indirect mechanisms. Our data suggest that both mechanisms can be envisaged in the context of muscular dystrophy. Interestingly, we did not observed any significant change in endogenous muscle levels of IL-6 between mdx and mdx/IL6 mice. Several evidences indicate that contracting muscle fibers themselves are a source of IL-6 mRNA and protein (28,33). Under physiologic conditions such as exercise, IL-6 is rapidly and markedly increased (79). Of note, the fact that the classical pro-inflammatory cytokines, TNFα and IL-1β, in general do not increase with exercise indicates that the cytokine cascade induced by exercise markedly differs from the cytokine cascade induced by infections or diseases (80). The muscle-induced IL-6 can act in an autocrine or paracrine manner, stimulating anabolic pathways associated with muscle growth, myogenesis and with regulation of energy metabolism (33). In contrast, under pathologic conditions, such as muscular dystrophy, cancer associated cachexia and other diseases, in which the plasma levels of IL-6 significantly increase, the anabolic muscle activity of IL-6 might be impinged and prevails the persistent and systemic activity of IL-6, which promote muscle wasting with the possible participation of other mediators (33).

Interestingly, we observed a significant up-regulation of the IL-6 receptor alpha, which mediates the IL-6 trans-signaling, in the muscle of 24-week-old mdx/IL6 mice compared with both wild-type and mdx littermates (Fig. 2). This suggests that the increased plasma levels of IL-6, in the mdx/IL6 mice, can directly target skeletal muscle, altering muscle homeostasis and/or indirectly, stimulating the activity of factors that contribute to myonecrosis, increased inflammation and altered regenerative mechanisms.

Of note, overexpression of IL-6 in a non-dystrophic animal model, although causes growth impairment, does not induce any active inflammation, necrosis, regeneration or fibrosis in muscle (34 and data not shown). The transgenic mice have normal food intake and a normal life span, with no peculiar susceptibility to infections (34). Thus, in the context of muscular dystrophy, increased circulating levels of IL-6 might synergize with other factors, creating an hostile microenvironment and exacerbating the dystrophic phenotype.

**Figure 6.**
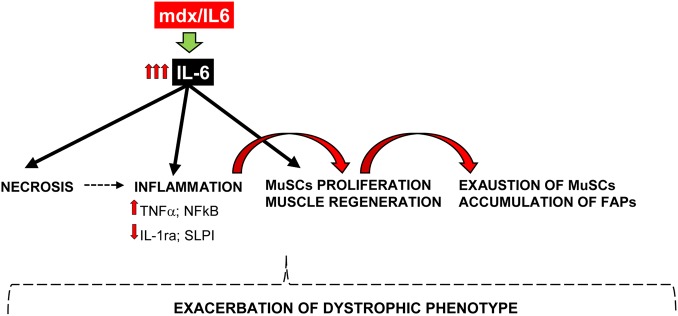
A schematic model depicting the effects of increased plasma levels of IL-6 on dystrophic muscle. Increased levels of IL-6 promote necrosis and inflammation, stimulating a continuous cycle of degeneration and regeneration, leading to exhaustion of MuSC and accumulation of fibro/adipocytic infiltration. All of these contribute to exacerbate the dystrophic phenotype.

In summary, we describe the generation of a new DMD mouse model that closely approximates the human disease and more faithfully recapitulates the disease progression in humans. Our work is consistent with a model (Fig. 6) in which increased circulating levels of IL-6 exacerbate the dystrophic phenotype, promoting muscle degeneration, inflammation, exhaustion of MuSCs and accumulation of FAPs. This study also indicate a potential therapeutic approach to counter the secondary symptoms caused by the primary loss of dystrophin, namely necrosis, inflammation and alteration in the regenerative mechanisms. Indeed, more recently, we demonstrated that inhibition of IL-6 activity, using an IL-6 receptor neutralizing antibody, conferred robustness to dystrophic muscle, significantly reduced necrosis, impeded the activation of a chronic inflammatory response, activated the circuitry of muscle differentiation and maturation, guaranteeing a functional homeostatic maintenance of dystrophic muscle (17). These findings were paralleled by inhibition of NFkB expression and with modulation of the inflammatory response, including down-regulation of TNFα and up-regulation of the anti-inflammatory mediators IL-1ra and SLPI, and with a down-regulation of Desmin, a marker of proliferating satellite cells (63,81). Interestingly, all of these factors were modulated in mdx/IL6 muscle, suggesting that IL-6 leads to changes in the dystrophic muscle environment, conditioning inflammatory responses and muscle repair.

Thus, our study promises to significantly advance our understanding of the pathogenic mechanisms that lead to muscular dystrophy and to provide the rationale for pharmacological treatment of DMD patients.

## Material and Methods

### Mice

Mdx mice (Jackson Laboratories) and IL-6 transgenic mice (34), which overexpress human IL-6 since birth, were used to generate the mdx/IL6 transgenic animals. IL-6 transgenic mice were previously generated using the NSE/hIL-6 construct, which carries the rat neurospecific enolase (NSE) promoter driving the expression of human IL-6 complementary DNA (34). Overexpression of IL-6 does not induce any neural alteration nor active inflammation, necrosis or fibrosis in muscle (34). The transgenic mice have normal food intake and hematic glucose as well as a normal life span, with no peculiar susceptibility to infections and no defects in the numbers of spleen T cells and B cells (34).

All animal experiments were approved by the ethics committee of Sapienza University of Rome-Unit of Histology and Medical Embryology and were performed in accordance with the current version of the Italian Law on the Protection of Animals.

### Force measurements

Twenty-four-week-old mice were sacrificed and force measurements of extensor digitorum longus (EDL) muscle were performed *ex vivo* as reported (82,83). To evaluate the decline of force, five tetanic stimulation were imposed to the muscle with a recovery period of 2 m between each of them. Drop of force was evaluated as the value of force developed during the last stimulation (F5) divided for the initial value of force (F1), expressed in percentage: F5/F1%.

### Treadmill tests

The treadmill tests were performed using the LE8700 Treadmill Control (Panlab sl). Twenty-four-week-old wild-type, mdx and mdx/IL6 mice were acclimated to treadmill running at 5 m/min for 5 min for two times. The mice ran for 30 min on a horizontal treadmill at a speed of 12 m/min, twice a week for 5 weeks. Distance (meter) was measured at 10, 20 and 30 min of running.

We used a motorized treadmill equipped with a gentle intensity shock grid. Animals who quit running were pushed by the moving treadmill belt onto a grid that delivers an electric foot shock; to escape the negative stimulus, the mice return to running on the belt. To avoid potential stress caused by the shock apparatus we also used an alternative gentle encouragement, by a human operator, that is a tongue depressor, coupled with sensitivity to the voluntary willingness to run on the part of the mouse (84). Of note, the results obtained with the two alternative methods of encouragement were comparable in all animals analysed.

### Evans blue staining and confocal microscopy

Intraperitoneal injection of Evans blue dye (EBD) (100 μl of 1% EBD per 10 g of body mass) was performed on a minimum of four animals/strain (24-week-old wild-type, mdx and mdx/IL6 mice), as described (85). Fluorescent fibers were viewed under an inverted microscope (Axioskop 2 plus; Carl Zeiss Microimaging, Inc.), and images were processed using Axiovision 3.1. and analysed using Scion Image 4.0.3.2. software. Confocal microscopy (Leica Laser Scanning TCS SP2) was used to analyse the total intensity of EBD fluorescence, which represent the full amount of fluorescence held within the entire z-axis of the series, in 4-week-old mdx and 24-week-old mdx whole diaphragm muscles. Approximately 160 optical sections, from at least three separate experiments, were analysed. The images were processed and analysed using LAF AF Lite software (Leica). Acquisition and analysis was performed in a blinded fashion, using coded slides.

### Histological analysis

Segments of diaphragm from a minimum of five animals/strain (24-week-old wild-type, mdx and mdx/IL6 mice) were embedded in tissue freezing medium and snap frozen in nitrogen-cooled isopentane. For general morphology, cryostat sections were stained with hematoxylin and eosin (H&E) according to standard protocols. Bright-field images were visualized using an Axioskop 2 plus (Carl Zeiss Microimaging, Inc.) and analysed using Scion Image 4.0.3.2. software to quantify: (i) the number of fibers with central nuclei per field and (ii) the mean of cross-sectional area (CSA) of centrally nucleated myofibers. For each tissue section analysed, a minimum of 300 muscle fibers were counted. Myeloperoxidase staining (SIGMA) was used to quantify the number of phagocytic cells per field. Histological evaluations were performed in a blinded manner, using coded slides.

### Protein extraction, Western blot analysis and ELISA

Diaphragm muscles from at least five animals/strain (wild-type, mdx and mdx/IL6 mice) were homogenized in modified lysis buffer [Tris–HCl, ph 7.5/20 mm, EDTA/2 mm, EGTA/2 mm, sucrose/250 mm, DTT/5 mm, Triton-X/0.1%, PMSF/1 mm, NaF/10 mm, SOV_4_/0.2 mm, cocktail protease inhibitors/1X (Sigma)]. Filters were blotted with antibodies against: Pax-7, Desmin, NFkBp65 (ser536), NFkB (Cell Signaling); GAPDH (Santa Cruz). Signals were captured by ChemiDoc-It^®^ Imaging System (UVP, LLC) and densitometric analysis was performed with VisionWorks^®^LS Image Acquisition and Analysis Software. Elisa assay was performed to detect murine and transgenic IL-6 using Quantikine^®^ Colorimetric Sandwich ELISAs (R&D Systems), according to manufacturer's protocol.

### RNA extraction and quantitative RT-PCR

Total RNA extraction was performed with tissue lyser (Qiagen) in TriRiagentTM (Sigma) and was reverse-transcribed using the QuantiTect Reverse Transcription kit (Qiagen). Quantitative PCR was performed on an ABI PRISM 7500 SDS (Applied Biosystems), using premade 6-carboxyfluorescein (FAM)-labeled TaqMan assays for HPRT1, IL-6, IL6Rα, gp130, SOCS3, Utrn, TNFα, IL1β, IL-1ra, SLPI, Pax-7, Desmin (Applied Biosystems). The relative level for each gene was calculated using the 2-DDCt method and reported as mean fold change in gene expression.

### Flow cytometry

Isolated diaphragm muscles were dissociated from wild-type, mdx and mdx/IL6 mice both mechanically, by mincing them into a coarse slurry with scissors, and enzymatically, using 0.2% collagenase type II (Sigma) in PBS for 90 min at 37°C (65). The isolated cells were then filtered through a 40 µm cell strainer (Falcon) and incubated with the following antibodies (10 ng/ml): CD31-PECy7, CD45-eFluor 450 (eBioscience); Sca-1-FITC (Macs), CD34-APC (BD Pharmigen) and α7integrin-PE (R&D Systems Inc.). A subsequent incubation with 7-Aminoactinomycin D (Sigma) was performed. Flow cytometry analysis was performed on a CyAN ADP DAKO and Summit 4.3 software was used for data acquisition and analysis.

### Statistical analysis

Statistical analysis was performed with GraphPad Prism Software. All data, if not differently specified, were expressed as mean ± SEM. Difference among groups, if not differently specified, were assessed with one-way ANOVA with a Bonferroni post test or Dunn's post Test, and between pairs with Mann–Whitney test or Student's *t*-test assuming two-tailed distributions. Statistical comparisons of survival were made with the long-rank test. Sample size was predetermined based on the variability observed in preliminary and similar experiments. All experiments requiring animal models were subjected to randomization based on litter. *P* < 0.05 is considered statistically significant.

## Funding

This work was supported by Telethon (grant no. GGP13013) and partly by Agenzia Spaziale Italiana (grant no. 2013-088-RO), PRIN (grant no. 2010R8JK2X) and AFM (grant no. 15652). Funding to pay the Open Access publication charges for this article was provided by Telethon.
